# Mean arterial pressure differences between cuff oscillometric and invasive blood pressure

**DOI:** 10.1038/s41440-025-02165-4

**Published:** 2025-03-03

**Authors:** Dean S. Picone, Martin G. Schultz, Matthew K. Armstrong, J. Andrew Black, Nathan Dwyer, Philip Roberts-Thomson, Thomas Weber, James E. Sharman

**Affiliations:** 1 School of Health Sciences, Faculty of Medicine and Health, University of Sydney, Sydney, NSW Australia; 2 Menzies Institute for Medical Research, University of Tasmania, Hobart, TAS Australia; 3 Department of Exercise Science, Falk College, Syracuse University, Syracuse, NY USA; 4 Royal Hobart Hospital, Hobart, TAS Australia; 5 Cardiology Department, Klinikum Wels-Grieskirchen, Wels, Austria

**Keywords:** Blood pressure determination, Pulse, Sphygmomanometers

## Abstract

Differences between automated cuff oscillometric blood pressure (BP) and invasive measurements are well described, but the causes are not fully understood. Automated BP devices record cuff oscillometric mean arterial pressure (MAP) as a key measurement step that is presumed to be accurate, but if not, could create error in cuff systolic (SBP) and diastolic BP (DBP) estimations. This has never been determined and was the aim of the study. Data from five studies with similar protocols were analysed (*N* = 262 patients undergoing coronary angiography, 61 ± 11 years, 65% male). Cuff oscillometric MAP was measured using five different models of automated cuff BP devices simultaneous to invasively measured MAP (fluid-filled or solid-state catheters). Cuff SBP and DBP were estimated by device-specific algorithms. Differences (∆) were calculated as cuff–invasive aortic BP. There were significant associations between ∆MAP and ∆SBP in four out of five devices (unstandardised β range = 0.42–1.04). The ∆MAP explained 6–52% of the variance in ∆SBP. In the same four devices, there were significant associations between ∆MAP and ∆DBP (unstandardised β range = 0.57–0.97) and ∆MAP explained 35–52% of the variance in ∆DBP. In conclusion, there are differences between cuff oscillometric MAP and invasive MAP which are associated with ∆SBP and ∆DBP. Further research is required to improve cuff oscillometric BP and greater transparency needed to understand algorithms used in these devices.

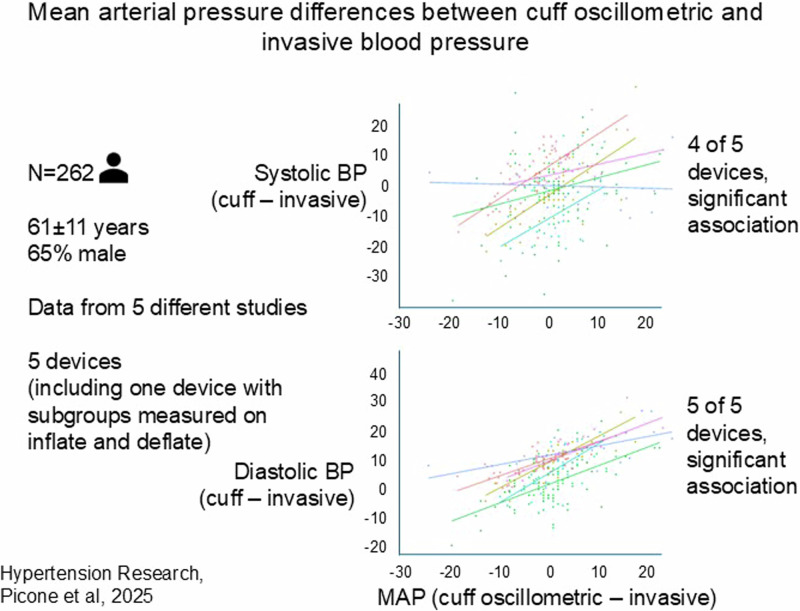

## Introduction

High blood pressure (BP) is the leading global risk factor for death and disability [1]. High-quality BP measurement is essential for correct hypertension diagnosis and management, and for this purpose, automated upper-arm cuff BP measurement devices are the recommended clinical standard [2–4]. These devices are supposed to emulate the BP values recorded by manual auscultation as an indirect measure of invasive BP [5]. However, meta-analyses of individual patient data have shown that automated cuff BP measurement devices have a significant level of both random and systematic error when compared with invasive BP measurement at the brachial or central aortic arteries [6]. Systematic error is associated with increasing age (cuff systolic BP underestimation and diastolic BP overestimation) and sex (underestimation of cuff systolic BP in women) and random error occurs between different devices [7, 8]. The cause of differences between automated cuff BP and invasive BP have not been fully determined but are essential to understand so that the quality of clinical BP measurement and risk stratification can be improved.

A critical measurement step that is common between different automated upper-arm cuff BP devices is the identification of the point of maximal amplitude on the oscillometric waveform envelope [9]. This feature is derived from the cuff deflation (or sometimes inflation) curve using proprietary algorithms (i.e. not publicly available) and is assumed to accurately represent the mean arterial pressure (MAP) based on intra-arterial confirmation in early studies of the 1970’s [10]. Once cuff oscillometric MAP has been identified, systolic and diastolic BPs can be estimated, again, using proprietary algorithms that are unique to each BP manufacturer (of which there are more than 450 internationally) [9, 11]. Nevertheless, it is thought that many of these algorithms derive systolic and diastolic BP values relative to the cuff oscillometric MAP on the oscillometric waveform. Therefore, any differences in the cuff oscillometric MAP to a reference standard may be associated with differences of consequent cuff systolic and diastolic BP values compared to the reference standard. This concept has never been explored and we sought to achieve this in the current study. We hypothesized there would be direct associations in the direction and magnitude of differences between cuff oscillometric MAP and intra-arterial MAP, and the consequent differences between systolic and diastolic BPs and intra-arterial measurements.

## Methods

### Study overview

Data are from five studies within the Invasive Blood Pressure Consortium with complete data relevant to this study aim. Each study recorded oscillometric MAP and invasive MAP measurements in addition to cuff and invasive aortic systolic and diastolic BPs among patients undergoing coronary angiography. The Invasive Blood Pressure Consortium was formed following a systematic review and individual participant data meta-analysis [6]. The rationale for using data from multiple studies was to understand if a consistent effect of differences in cuff oscillometric MAP compared with invasive MAP was observed across different automated cuff BP devices, each with unique proprietary algorithms. Although it would have been preferable to test multiple devices on the same patient, this was not feasible within the busy clinical centres where data was recorded.

All recordings were made under baseline hemodynamic conditions. In all studies the cuff and invasive aortic BP were measured contemporaneously. In three studies, cuff BP and invasive aortic BP was precisely simultaneous (one unpublished) [12, 13], in one study invasive aortic BP was recorded immediately prior to the cuff BP [14], and in one study cuff BP was recorded immediately prior to invasive aortic BP [15]. Exclusion criteria across all studies included valvular heart disease and arrhythmia, on the basis that these conditions could influence the quality of the cuff and invasive aortic BP recordings. The rationale for comparing cuff and invasive aortic BP is that the cuff BP was originally intended to measure aortic BP as a marker of BP exposure of the central organs [16], and most automated cuff BP devices provide a closer estimate of aortic BP than upper arm BP [6, 17]. Each research site obtained local ethics approval and the data for this investigation was merged with the approval of all data custodians in accordance with Tasmanian Health and Medical Human Research Ethics Committee (reference: H0015048).

### Automated (oscillometric) cuff BP measurements

Participants were fitted with appropriately sized cuffs in all studies. In two studies, duplicate cuff BP measurements were taken and averaged for analysis (one unpublished) [12]. In the other three studies a single cuff measurement was taken [13–15]. The cuff BP devices used in the measurements were the Oscar 2 (SunTech Medical Inc, Morrisville, NC), Mobil-o-graph (IEM GmbH, Aachen, Germany) [12], Colin Medical Instruments (San Antonio, TX) [13], Welch Allyn NIBP module (Skaneateles Falls, NY) within a PulseCor R7 device (Auckland, New Zealand) [15], and PAR UP NIBP within the Uscom BP+ device (Sydney, Australia, unpublished; PAR UP NIBP is now superseded by a new non-invasive module in the BP+ device). The PAR UP NIBP measurement device recorded during inflation in 22 participants and during deflation in 12 participants. Four of the five devices had evidence of passing validation testing compared with an auscultatory BP reference standard [18–21].

### Intra-arterial BP measurements

Catheters were positioned in the ascending aorta in all studies, with arterial access via either the femoral or radial arteries. Fluid-filled catheters were used in four studies (one unpublished) [13–15], and solid-state catheters in one study [12]. Recording periods for the invasive measurements in each study varied and included 5 s [14], 10–15 s [15], an average over several respiratory cycles (which equates to approximately 15 s) [13], period of measurement, either from commencement of cuff inflation to maximal cuff inflation (measure on inflate) or from maximal cuff inflation to full deflation (measure on deflate; unpublished study) and 3–4 min [12]. Invasive aortic MAP was measured via integration of the aortic pressure waveform in all studies. Measurements were recorded with the catheter and pressure transducer positioned mid-chest to negate effects of hydrostatic pressure.

### Statistical analysis

BP data are reported as mean difference ± standard deviation. Differences (∆) in cuff (oscillometric) BP compared to invasive BP were calculated as cuff minus invasive aortic BP for all comparisons and assessed using paired T-tests. The mean absolute difference, which is calculated by ignoring the direction of the difference between the two measurements. Associations between ∆MAP (cuff oscillometric MAP – invasive MAP) and ∆systolic BP or ∆diastolic BP were quantified by linear regression. Multivariable linear regression was also performed with age, sex, body mass index and heart rate as covariables, except for the PAR UP NIBP due to insufficient sample size for adjustment. Additionally, cuff oscillometric MAP, systolic BP or diastolic BP were separately included in the base model to avoid collinearity. *P* < 0.05 was considered statistically significant.

## Results

### Participants

There were minor differences across the various studies for the participant characteristics, but in general, participants were middle-to-older age, mostly male and had an average body mass index >25 kg/m^2^ (Table 1). Heart rate was between 66 and 74 beats per minute, on average, for the individual studies.

**Table 1:** Participant characteristics across different studies and cuff blood pressure measurement devices

Variable	Schultz et al. [14] Device: Oscar 2	Costello et al. [15] Device: Welch Allyn NIBP module^a^	Smulyan et al. [13] Device: Colin Medical Instruments	Weber et al. [12] Device: Mobil-o-graph	Picone et al., unpublished. Device: PAR UP NIBP^b^	
					Inflate	Deflate
Sample size	57	41	100	30	22	12
Age, years	63 ± 10	62 ± 11	60 ± 12	59 ± 11	65 ± 8	31 ± 13
Male sex, %	42 (74)	26 (63)	49 (49)	26 (87)	17 (81)^c^	5 (83)^d^
Height, cm	172 ± 9	171 [160 to 175]	169 ± 10	174 ± 6	174 ± 12	173 ± 7
Weight, kg	86 ± 16	85.5 ± 20	87.7 ± 23	85.5 ± 15	91.7 ± 19	91.2 ± 18
Body mass index, kg/m^2^	29 ± 5	30.1 ± 6	30.7 ± 8	28.1 ± 5	30.4 ± 5	30.7 ± 7
Heart rate, beats/min	68 ± 12	74 [66 to 82]	71 ± 13	70 ± 11	68 ± 13	62 ± 9

Data are mean ± standard deviation or median [interquartile range]

NIBP, non-invasive blood pressure

^a^Within PulseCor R7 which is no longer available

^b^Within Uscom BP+, the version of the device used in this study has now been superseded and is no longer available

^c^Sex not recorded in one participant

^d^Sex not recorded in 5 participants

### Cuff oscillometric and invasive aortic BP measurements

Cuff MAP, systolic BP and diastolic BP values from all studies were 96 ± 15, 132 ± 22 and 76 ± 12 mmHg. The average invasive aortic MAP, systolic BP and diastolic BP values were 95 ± 14, 132 ± 24 and 70 ± 12 mmHg. BP values from the individual studies are reported in Table 2. The mean ∆MAP varied between studies from −2.6 to +9.5 mmHg. The average ∆systolic BP were between −8.8 to +5.1 mmHg whilst ∆diastolic BP were +2.0 to +14.2 mmHg. Mean absolute differences in cuff MAP ranged from 3.3 to 10.1 mmHg, in cuff systolic BP ranged from 5.5 to 11.8 mmHg and in cuff diastolic BP ranged from 5.0 to 14.5 mmHg.

**Table 2:** Cuff oscillometric blood pressure and invasive aortic blood pressure across different studies and measurement devices

Variable	Schultz et al. [14] Device: Oscar 2	Costello et al. [15] Device: Welch Allyn NIBP module^a^	Smulyan et al. [13] Device: Colin Medical Instruments	Weber et al. [12] Device: Mobil-o-graph	Picone et al., unpublished. Device: PAR UP NIBP^b^	
					Inflate	Deflate
Cuff MAP	86 ± 10	94 ± 12	100 ± 17	103 ± 10	101 ± 16	88 ± 12
Aortic MAP	89 ± 13	92 ± 13	99 ± 11	102 ± 9	92 ± 10	85 ± 6
Cuff-aortic MAP	−2.6 ± 6.1, *p* = 0.0024	1.5 ± 6.7, *p* = 0.15	0.8 ± 7.2, *p* = 0.29	1.4 ± 5.5, *p* = 0.16	9.5 ± 8.6, *p* < 0.001	3.0 ± 9.0, *p* = 0.27
Mean absolute error MAP	3.3 [1.5–7.5]	5.0 [2.0–8.0]	3.5 [2.0–7.3]	3.5 [2.1–6.9]	10.1 [3.4–16.1]	5.1 [2.4–8.8]
Cuff SBP	128 ± 18	130 ± 18	139 ± 26	129 ± 16	130 ± 19	119 ± 14
Aortic SBP	124 ± 22	131 ± 19	139 ± 27	138 ± 19	130 ± 22	114 ± 14
Cuff-aortic SBP	4.4 ± 8.7, *p* = 0.0003	−0.9 ± 11, *p* = 0.61	−0.8 ± 11, *p* = 0.29	−8.8 ± 10, *p* < 0.0001	0.1 ± 8.0, *p* = 0.96	5.1 ± 6.5, *p* = 0.020
Mean absolute error SBP	6.8 [2.9–12.0]	7.0 [4.0–10.0]	6.0 [2.0–11.0]	11.8 [5.3–17.0]	5.5 [2.9–8.1]	6.5 [3.7–8.8]
Cuff DBP	74 ± 10	77 ± 10	75 ± 14	81 ± 8	83 ± 13	74 ± 11
Aortic DBP	65 ± 10	66 ± 11	73 ± 13	74 ± 7	68 ± 11	63 ± 9
Cuff-aortic DBP	8.8 ± 5.1, *p* < 0.0001	10.3 ± 9.0, *p* < 0.0001	2.0 ± 7.4, *p* = 0.0070	6.7 ± 7.3, *p* < 0.0001	14.2 ± 6.2, *p* < 0.0001	11 ± 7, *p* = 0.0001
Mean absolute error DBP	9.2 [5.9–11.0]	10.0 [7.0–13.0]	5.0 [2.0–8.0]	7.3 [3.8–10.8]	14.5 [10.4–17.3]	11.7 [6.0–15.6]

Data are mean ± standard deviation or median [interquartile range]. The units in all pressure rows are mmHg

*NIBP* non-invasive blood pressure, *SBP* systolic blood pressure, *DBP* diastolic BP, *MAP* mean arterial pressure

^a^Within PulseCor R7 which is no longer available

^b^Within Uscom BP+, the version of the device used in this study has now been superseded and is no longer available

### Association between ∆MAP and ∆systolic BP

**Fig. 1:**
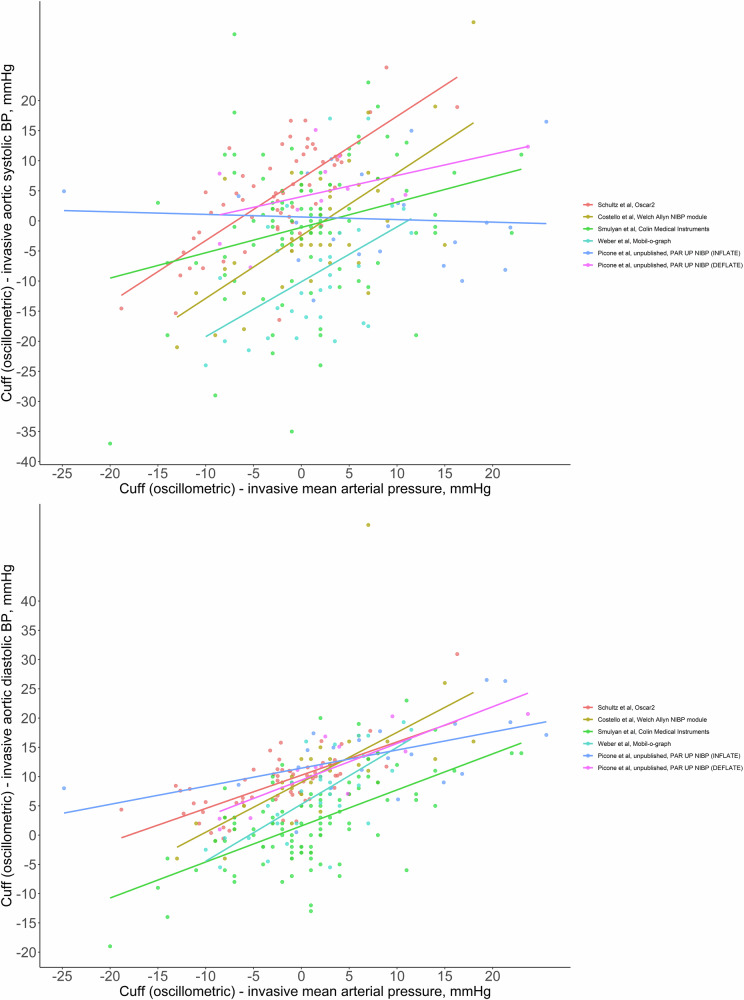
Relationship between the difference in cuff oscillometric mean arterial pressure and invasive mean arterial pressure (x-axis both plots) and the difference in cuff and invasive systolic blood pressure (systolic BP; y-axis top plot) or the difference in cuff and invasive diastolic BP (y-axis bottom plot) recorded from five different automated cuff oscillometric BP devices (including one device with measurement on inflate and measurement on deflate capacity) compared with invasive aortic BP. Each of the six different devices/measurement modalities is represented with a different colour for the individual data points and trend lines. All differences were calculated as cuff minus invasive aortic BP. The Welch Allyn non-invasive BP module was within the PulseCor R7 device which is no longer available. The PAR UP non-invasive BP module was within the Uscom BP+ and the version of the device used in this study has now been superseded and is no longer available

The ∆MAP was associated with ∆systolic BP in the pooled dataset (Fig. 1). In four out of five individual devices there was an association between the ∆MAP and ∆systolic BP (Table 3). In the four devices that had a significant association between the ∆MAP and ∆systolic BP, the strength of the relationships were variable (unstandardised β (95% CI): 0.42 (0.12–0.72) to 1.04 (0.66–1.42)). For the fifth device, measurement on deflate showed a similar, but non-significant relationship between ∆MAP and ∆systolic BP (unstandardised β (95% CI): 0.35 (−0.09–0.79)), whereas there was no association for measure on inflate (unstandardised β (95% CI): −0.042 (−0.37–0.29)). In the four devices with a significant association between ∆MAP and ∆systolic BP, the ∆MAP explained between 6% and 52% of the variance in the ∆systolic BP, in contrast to the fifth device (16% for measure on deflate and 0% for measure on inflate; adjusted R^2^).

**Table 3:** Univariable and multivariable analysis of associations between ∆MAP and ∆systolic BP or ∆diastolic BP

Variable	Schultz et al. [14] Device: Oscar 2	Costello et al. [15] Device: Welch Allyn NIBP module^a^	Smulyan et al. [13] Device: Colin Medical Instruments	Weber et al. [12] Device: Mobil-o-graph	Picone et al., unpublished. Device: PAR UP NIBP^b^	
Univariable analysis						
Dependent variable	Predictor variable is ∆MAP (cuff oscillometric– invasive aortic MAP)					
					Inflate	Deflate
∆Systolic BP	β = 1.03 (0.77–1.30) Adjusted R^2^ = 0.52	β = 1.04 (0.66–1.42) Adjusted R^2^ = 0.43	β = 0.42 (0.12–0.72) Adjusted R^2^ = 0.063	β = 0.91 (0.27–1.56) Adjusted R^2^ = 0.20	β = −0.042 (−0.37–0.29) Adjusted R^2^ = −0.05	β = 0.35 (−0.09–0.79) Adjusted R^2^ = 0.16
∆Diastolic BP	β = 0.57 (0.41–0.73) Adjusted R^2^ = 0.47	β = 0.85 (0.52–1.19) Adjusted R^2^ = 0.39	β = 0.62 (0.45–0.78) Adjusted R^2^ = 0.35	β = 0.97 (0.62–1.32) Adjusted R^2^ = 0.52	β = 0.31 (0.09–0.52) Adjusted R^2^ = 0.28	β = 0.62 (0.34–0.91) Adjusted R^2^ = 0.68
Multivariable analyses						
Dependent variable	Predictor variable is ∆MAP (cuff – invasive aortic MAP). Adjusted for age, sex, heart rate, body mass index and invasive aortic MAP.					
∆Systolic BP	β = 1.05 (0.73–1.37) Adjusted R^2^ = 0.61	β = 1.01 (0.73–1.37) Adjusted R^2^ = 0.61	β = 0.59 (0.31–0.89) Adjusted R^2^ = 0.26	β = 1.13 (0.63–1.62) Adjusted R^2^ = 0.59	Multivariable adjustment not performed due to low sample sizes in inflation/deflation sub-groups.	
∆Diastolic BP	β = 0.88 (0.72–1.04) Adjusted R^2^ = 0.71	β = 0.83 (0.43–1.23) Adjusted R^2^ = 0.51	β = 0.62 (0.44–0.80) Adjusted R^2^ = 0.33	β = 0.80 (0.51–1.08) Adjusted R^2^ = 0.72		
Dependent variable	Predictor variable is cuff – invasive aortic MAP (MAP error). Adjusted for age, sex, heart rate, body mass index and invasive aortic systolic BP.					
∆Systolic BP	β = 0.93 (0.63–1.23) Adjusted R^2^ = 0.62	β = 0.94 (0.59–1.29) Adjusted R^2^ = 0.67	β = 0.68 (0.40–0.96) Adjusted R^2^ = 0.33	β = 1.09 (0.64–1.55) Adjusted R^2^ = 0.64	Multivariable adjustment not performed due to low sample sizes in inflation/deflation sub-groups.	
∆Diastolic BP	β = 0.85 (0.71–0.99) Adjusted R^2^ = 0.71	β = 0.83 (0.48–1.20) Adjusted R^2^ = 0.52	β = 0.61 (0.43–0.80) Adjusted R^2^ = 0.33	β = 0.82 (0.53–1.11) Adjusted R^2^ = 0.70		

Data are β coefficient (95% confidence interval)

*NIBP* non-invasive blood pressure, *BP* blood pressure, *MAP* mean arterial pressure, ∆*MAP* cuff oscillometric MAP – invasive MAP, ∆*Systolic BP* cuff systolic BP – invasive systolic BP, ∆*Diastolic BP* cuff diastolic BP – invasive diastolic BP

^a^Within PulseCor R7 which is no longer available

^b^Within Uscom BP+, the version of the device used in this study has now been superseded and is no longer available

### Association between ∆MAP and ∆diastolic BP

The ∆MAP was associated with the ∆diastolic BP in the pooled dataset (Fig. 1). In all five devices, there were significant associations between the ∆MAP and ∆diastolic BP, including for measurement on inflate and deflate in the fifth device (Table 2). Across the individual devices, the strength of the associations between the ∆MAP and ∆diastolic BP were variable (unstandardised β and 95% CI: 0.31 (0.09–0.52) to 0.97 (0.62–1.32)). The ∆MAP explained between 28% and 52% of the variance in the ∆diastolic BP (adjusted R^2^).

The ∆MAP remained significantly associated with ∆systolic BP and ∆diastolic BP after adjustment for age, sex, heart rate, body mass index and invasive BP (aortic systolic BP, aortic diastolic BP, aortic MAP or aortic pulse pressure each added to models separately; Table 3).

## Discussion

To our knowledge, this is the first study to examine if the difference between automated cuff oscillometric MAP and invasive MAP may relate to the difference in cuff systolic BP and diastolic BP compared with invasive measurement. This was determined using an invasive reference standard across five unique automated cuff BP measurement devices. The main findings were that ∆MAP was associated with ∆systolic BP in four of five devices, whereas ∆MAP was associated with ∆diastolic BP in all five devices. The strength of the associations between ∆MAP and ∆systolic or ∆diastolic BP, and magnitude of the differences were device specific. These data highlight that equivalence between cuff oscillometric MAP and invasive MAP cannot be assumed, and this could be a significant factor contributing to ∆systolic or ∆diastolic BP. Efforts to improve oscillometric BP measurement methods are needed, as well as greater transparency of the BP estimation algorithms that are used in commercially available devices.

The exact approach used by automated BP devices to determine MAP and subsequently estimate systolic BP and diastolic BP is unknown. However, it is well described that the peak amplitude of the oscillometric waveform envelope is used to identify MAP in automated cuff BP devices. To generate the oscillometric waveform envelope, a series of arterial pulses are captured during cuff deflation (or inflation in some devices). These pulses are put through a high pass filter, and signal processing applied, using device-specific methods to produce a waveform envelope. Each of these steps could influence the final shape of the envelope that is generated, and thus accurate identification of peak amplitude and MAP [9, 22]. For example, a waveform envelope with a clearly defined (single) peak amplitude may be less susceptible to measurement error than a broad, flatter waveform envelope with a poorly defined peak amplitude. This hypothesis is well demonstrated in the work of Amoore and colleagues (Figures 2–4 of their manuscript) [23], who showed that the best accuracy of oscillometric systolic BP and diastolic BP occurred when the waveform envelopes have a distinct peak amplitude (albeit noting that their reference standard was auscultatory systolic BP and diastolic BP rather than invasive BP as we used in this current study) [24]. More recent work has illustrated that asymmetrical, skewed oscillometric waveforms are more likely to occur with increasing age [25], in people with high BP [24, 25], and in hypertensive disorders of pregnancy [26]. These features may lead to less accurate BP measurement in these populations, especially when fixed-ratios or slope-based algorithms are used to estimate systolic BP and diastolic BP from the oscillometric waveform relative to MAP.

The findings that ∆MAP was associated with ∆systolic BP were consistent across 4 of the 5 devices (and all 5 devices for ∆diastolic BP) tested in this study. For the fifth, inconsistent device, this had a function to measure on inflation or deflation and both were used on different participant sub-groups. There was a small sample size for the sub-group with measurement on deflation, but still a trend toward an association between ∆MAP and ∆systolic BP, which was consistent with the pattern for the other four devices. On the other hand, for the measure on inflate sub-group there was no association between ∆MAP and ∆systolic BP, which may indicate use of different algorithms between inflation and deflation modes, or some other factors associated with different measurement conditions (e.g. longer arterial occlusion during deflation). Overall, these findings may be generalizable across different oscillometric devices, in particular those that measure BP when the cuff is deflating. However, there are several thousand unique oscillometric BP devices available on the market [11] each using a proprietary algorithm for BP estimation, and it is not feasible to test all of these devices against an invasive BP reference standard.

Some recent examples of possible causes of poor quality measurement by oscillometric BP are the methods for generating the oscillometric waveform envelope and disturbances to cuff pulse recordings. A study of 73 healthy participants (51 ± 18 years, 63% female) tested different methods to generate the oscillometric waveform envelope and consequent accuracy of systolic BP and diastolic BP [22]. There was different accuracy of systolic BP and diastolic BP that was particularly evident when participants were split into tertiles of age, with lowest accuracy among the participants 41-62 years of age, the middle age tertile. The authors also observed that different methods to generate the oscillometric waveform envelope resulted in changes to the maximal amplitude but could not determine the extent to which this influenced the accuracy of MAP due to the absence of a ground truth measurement of MAP. Other recent work among 30 people (65 ± 10 years, 30% female) with a clinical indication for 24-hour ambulatory BP monitoring tested the influence of pulse disturbances during recording of the cuff deflation curve, caused by arrhythmia and movement [27]. Compared to recordings free of pulse disturbances, those with pulse disturbances (26% of all measurements) were on average 6.3 mmHg higher for daytime systolic BP. These pulse disturbances may lead to measurement errors due to suboptimal generation of the oscillometric envelope. Thus, improved artefact detection on oscillometric devices might help to improve measurement accuracy.

Recent studies have described significant differences between oscillometric BP compared with invasive BP. In an analysis of 31 studies and 2013 participants [28], oscillometric systolic BP overestimated invasive aortic systolic BP in children and underestimated in older people, whereas oscillometric diastolic BP progressively overestimated invasive diastolic BP with increasingly older age. There also appear to be sex differences such that for the same level of invasive aortic systolic BP, women have a cuff systolic BP that is ~4 mmHg lower than men [8]. Similar data has been observed in a separate study [29], however, the sex difference was largely mediated by height. Altogether, the evidence from recent studies and the present findings show that there is a need for more effort to improve the measurement of BP using the oscillometric method. This may be achieved by using arterial waveform information recorded during cuff inflation and deflation, which shows differences according to sex and age [30]. Innovations to achieve this could be accelerated by partnerships with BP device manufacturers. The accuracy of oscillometric BP is relevant to current best practice BP monitoring but will also be important for next-generation wearable technologies that use it as the calibration BP value.

### Limitations

This study cannot determine whether the ∆MAP causes the ∆systolic BP and ∆diastolic BP. Nevertheless, based on the measurement principles of oscillometric BP devices, this is a reasonable hypothesis. The data were collected in patients with a clinical indication for coronary angiography and therefore may not be generalisable beyond this population. Five different studies with slightly different protocols were used in the analysis, thus differences between cuff and invasive BP could be caused by factors such as acute BP fluctuations, vascular responses to cuff inflation and whether BP recordings were captured during inflation or deflation. There were only five oscillometric devices used in this analysis, despite knowledge that thousands of unique devices are available on the market [11]. Four of the five devices had been validated compared with an auscultatory BP reference standard. Despite these limitations, the broadly consistent results across the five devices suggests the findings may be generalisable to other oscillometric devices. Each study included in the analysis performed measurements using an appropriately sized cuff. However, arm circumference, specific cuff sizes and the precise methods for cuff selection used in each individual study were not available. Whether the association of ∆MAP with ∆systolic BP and ∆diastolic BP is explainable by variation in arm circumference is unknown and should be an area of future investigation.

## Conclusions

The difference between oscillometric MAP and invasive MAP was associated with the difference in cuff systolic BP and diastolic BP compared with invasive BP. Efforts to improve oscillometric BP measurement methods should be prioritised. Advocacy to increase transparency of oscillometric algorithms used in BP devices is also needed to understand the way that BP is measured and enable open-source availability of deidentified cuff oscillometric traces to accelerate research on the topic.
